# Comparing the efficacy of thyroglobulin and thyroglobulin/ thyroid-stimulating hormone ratio models in predicting a successful response to radioactive iodine therapy

**DOI:** 10.1186/s12902-022-01261-7

**Published:** 2023-01-20

**Authors:** Yanli Ju, Lihua Wang, Fang Cheng, Fengyan Huang, Xueyu Chen, Qingqing Song, Juan Xiao, Xiaolu Zhu, Hongying Jia

**Affiliations:** 1 Department of Epidemiology and Health Statistics, School of Public Health, Cheeloo College of Medicine, Shandong University, No. 44 Wenhua West Road, Jinan, Shandong 250012 China; 2 Center of Evidence-Based Medicine, the Second Hospital, Cheeloo College of Medicine, Shandong University, No. 247 Beiyuan Street, Jinan, Shandong 250033 China; 3 Department of Nuclear Medicine, the Second Hospital, Cheeloo College of Medicine, Shandong University, No. 247 Beiyuan Street, Jinan, Shandong 250033 China

**Keywords:** Differentiated thyroid cancer, Thyrotropin, Thyroglobulin, Radioactive iodine therapy

## Abstract

**Background:**

The thyroglobulin (Tg)/ thyroid-stimulating hormone (TSH) ratio has manifested to be a reliable marker for predicting prognosis in patients with differentiated thyroid carcinoma (DTC). The objective of this study was to compare the efficacy of Tg and Tg/TSH ratio models in predicting a successful response to radioactive iodine therapy.

**Methods:**

One thousand six hundred forty-two DTC patients receiving ^131^I radiotherapy were finally enrolled in this retrospective study. The patients were divided into a training set (*n* = 973) and a validation set (*n* = 669) by the patient consultation time (July 2019). A receiver-operating characteristic curve was constructed for Tg and the Tg/TSH ratio to establish their cutoffs. Then, the variables were screened by univariate logistic regression and incorporated into logistic prediction models by stepwise regression, where Tg/TSH was excluded from model 1 and Tg was excluded from model 2.

**Results:**

In 1642 enrolled DTC patients, the first ^131^I radiotherapy had an excellent response in 855 patients. The cut-offs for Tg level and Tg/TSH ratio were 3.40 ng/ mL [area under the curve (AUC): 0.789] and 36.03 ng/mIU (AUC: 0.788), respectively. In addition, the AUC of the model including Tg was higher than that of the model including Tg/TSH in both the training set (0.837 vs 0.833) and the testing set (0.854 vs 0.836).

**Conclusions:**

Both Tg and Tg/TSH ratios could be considered predictors of the effects of the first ^131^I ablative therapy. However, the prediction model including Tg performed better than the model including Tg/TSH.

## Introduction

Thyroid cancer ranked 9th for incidence in 2020 and is the most common endocrine malignancy worldwide [1, 2]. Of all the thyroid carcinoma, differentiated thyroid cancer (DTC) accounts for nearly 90% [3]. The standard treatment for DTC is total or near-total thyroidectomy followed by radioactive iodine (RAI) therapy and thyroid-stimulating hormone (TSH) suppression with levothyroxine (L-T4). With this treatment approach, the majority of patients with DTC could get improved disease-free survival and long-term prognosis [4]. However, in some patients, the persistent disease develops after initial treatment, or cancer recurrence is detected during follow-up.

The RAI could destroy the remaining residual tissue or metastatic tissue susceptible to radioiodine uptake, and is crucial to the treatment of thyroid cancer [5]. Its results affect the prognosis of patients with DTC and guide the decision-making process leading to RAI treatment. Thyroglobulin (Tg) has been demonstrated to be critical in predicting therapeutic response to RAI [6–8]. Since the production of Tg could be stimulated by the TSH, Tg values may be affected by the TSH values of pre-ablative patients. For this reason, the relationship between the Tg/TSH ratio and the outcome of the first ^131^I radiotherapy has been extensively studied [9–11]. However, few studies have compared the predictive model capability to the RAI ablation therapy of Tg and Tg/TSH.

This study aimed to assess the factors affecting the outcome of the first ^131^I radiotherapy in patients with DTC and compare the efficacy of Tg and Tg/TSH ratio models in predicting a successful response to radioactive iodine therapy.

## Material and methods

### Study patients

**Fig. 1:**
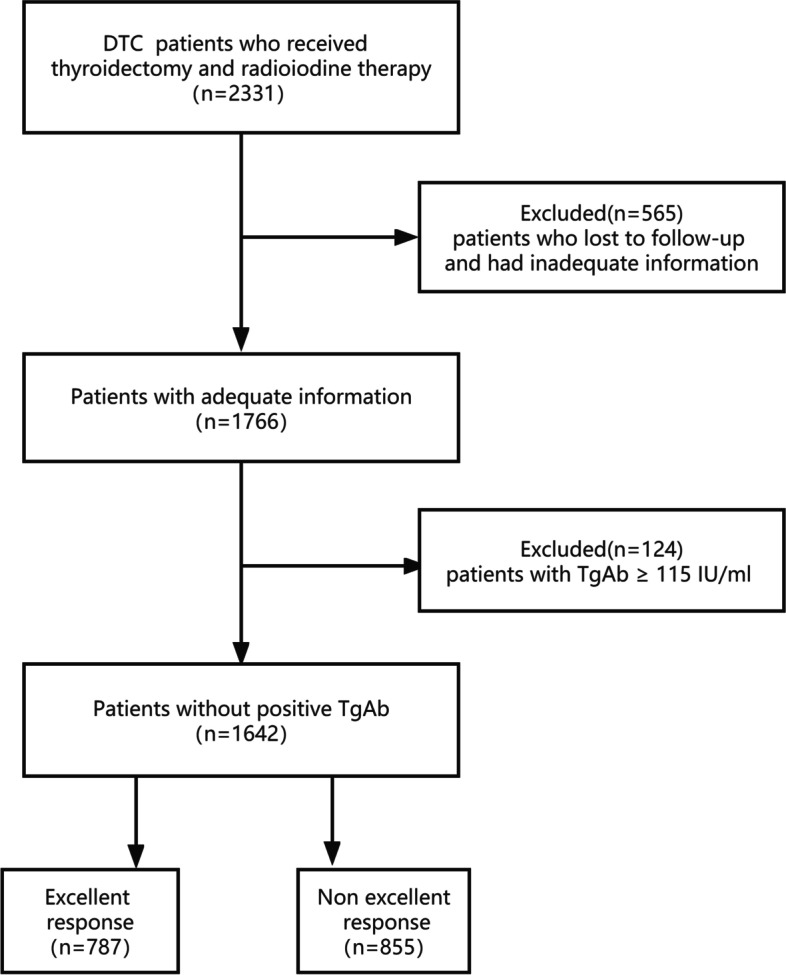
The flow chart of the study

This retrospective study reviewed the clinical records of 2331 DTC patients treated consecutively at our institution between September 2015 and September 2020. Patients’ data were extracted from the clinical electronic medical record system of the Second Hospital of Shandong University. Inclusion criteria were as follows:(1) adult patients with anatomopathological DTC diagnosis, (2) patients who received total or near-total thyroidectomy, and (3) patients who received ^131^I treatment followed. Exclusion criteria were as follows:(1) patients who presented thyroglobulin antibody (TgAb) ≥ 115 IU/ml, (2) patients who lost to follow-up and had inadequate information (Fig. 1). After the inclusion and exclusion criteria screening, 1642 patients were finally enrolled in this study.

Patient data extracted from the clinical electronic medical record system was identified such that all private information was not included. The informed consent was waived by the Institutional Review Board of the Second Hospital of Shandong University [KYLL-2018(LW)013]. All procedures complied with the Declaration of Helsinki for research involving human subjects.

### Treatment and examination

All patients underwent L-T4 withdrawal or no L-T4 therapy after total or near-total thyroidectomy for 3–4 weeks, accompanied by a low-iodine diet (< 50 ugs per day) for 1–2 weeks. When prepared well, a treatment dose from 1.11 GBq (30 mCi) to 5.55 GBq (150 mCi) of ^131^I was given on an empty stomach according to their tumor stage and recurrence risk stratification. The following tests were completed within three days before ^131^I treatment, including TSH, free thyroxine, free triiodothyronine, Tg, and TgAb.

### Evaluation of the therapeutic effect of ^131^I radiotherapy

Approximately six months after the ^131^I treatment, the curative efficacy was evaluated. The risk assessment after the initial ^131^I treatment was analyzed according to the 2015 American Thyroid Association (ATA) guideline, and the clinical therapeutic results were divided into four categories: Excellent Response (ER), Indeterminate Response (IDR), Biochemical Incomplete Response (BIR), and Structural Incomplete Response (SIR). The analysis classified the four response-to-therapy categories into ER and non-excellent response (NER), the latter including IDR, BIR, and SIR.

### Statistical analysis

Continuous variables were reported as mean and standard deviation or median values and quartiles, while categorical variables were reported as numbers and percentages. Between groups, differences were assessed with the student t-test (and Mann–Whitney U test) or the chi‑square test (categorical variables). The patients were divided into a training set (*n* = 973) and a validation set (*n* = 669) by the patient consultation time (July 2019). A receiver-operating characteristic (ROC) curve was constructed for Tg and the Tg/TSH ratio to establish their cutoffs. Then, univariate logistic regression was used to screen the factors associated with RAI therapy efficacy. The screened variables were incorporated into logistic prediction models by stepwise procedure, where Tg/TSH was excluded from model 1 and Tg was excluded from model 2. The performance of the models was evaluated by the AUC, accuracy, and specificity. The accuracy and clinical benefit of the model were assessed by the calibration curves and decision curves, separately. All tests were two-sided, and a *P*-value < 0.05 was considered statistically significant. The statistical analysis was performed on the software package R (version 4.1.1).

## Results

### Characteristics of patients

Of the 1642 DTC patients receiving ^131^I thyroid remnant ablation, the mean age at diagnosis was 43.69(± 11.50) years, and the majority (*n* = 1162; 70.8%) of DTC were female. Characteristics of study subjects are presented in Table 1.

**Table 1:** Comparison of baseline information for the training and testing sets

	Level	Overall(1642)	training set (973)	testing set (669)	*P*-value
Age *		43.69 (11.50)	43.56 (11.45)	43.89 (11.59)	0.569
Gender (%)	Male	480 (29.2)	286 (29.4)	194 (29.0)	0.906
	Female	1162 (70.8)	687 (70.6)	475 (71.0)	
Pathology type (%)	Papillary carcinoma	1633 (99.5)	969 (99.6)	664 (99.3)	0.571
	Follicular carcinoma	9 (0.5)	6 (0.7)	5 (0.7)	
Invasion (%)	No	1145 (69.7)	757 (77.8)	388 (58.0)	< 0.001
	Yes	497 (30.3)	216 (22.2)	281 (42.0)	
Metastasis number #		3 [2, 7]	3 [1, 6]	4 [2, 7]	< 0.001
Benign lesion (%)	No	696 (42.4)	427 (43.9)	269 (40.2)	0.153
	Yes	946 (57.6)	546 (56.1)	400 (59.8)	
TNM stage (%)	I	1382 (84.2)	827 (85.0)	555 (83.0)	0.298
	II-III	260 (15.8)	146 (15.0)	114 (17.0)	
ATA risk stratification (%)	Low risk	402 (24.5)	257 (26.4)	145 (21.7)	0.033
	Intermediate-high risk	1240 (75.5)	716 (73.6)	524 (78.3)	
Tg (ng/mL) #		2.2 [0.6,7.1]	2.2[0.5,7.4]	2.2[0.6,7.0]	0.531
Tg/TSH (ng/mIU) #		24.7 [5.8,83.7]	26.5[6.2,87.9]	21.9[5.2,75.7]	0.177
conclusion (%)	ER	787 (47.9)	470 (48.3)	317(47.4)	0.752
	NER	855 (52.1)	503 (51.7)	352 (52.6)	

*TSH* Thyroid stimulating hormone, *Tg* Thyroglobulin, *TNM stage* Tumor-node-metastasis stage of patients, *ER* Excellent response, *NER* Non-excellent response

^*^, (mean (SD); #, median [first quartile, third quartile]

Statistical tests: (%), the chi-square test; *, the student t-test; #, the Mann–Whitney U test. Statistical significance: *p* < 0.05

### Comparison between excellent response and non-excellent response groups

In 1642 enrolled DTC patients, the first ^131^I thyroid remnant ablation had an excellent response in 787 patients and had an incomplete answer in the remaining 855 patients, with a rate of excellent response of 52.1%. The differences in gender, metastasis number, benign lesion, ATA risk stratification, Tg and Tg/TSH ratio between patients with successful and unsuccessful ablation were statistically significant. (*P* < 0.05) (Table 2).

**Table 2:** Clinical pathologic characteristics of the cohort

	Level	Overall(1642)	ER(787)	NER(855)	*P*-value
Age*		43.69 (11.50)	43.36 (11.11)	44.00 (11.86)	0.255
Gender (%)	Male	480 (29.2)	169 (21.5)	311 (36.4)	< 0.001
	Female	1162 (70.8)	618 (78.5)	544 (63.6)	
Pathology type (%)	Papillary carcinoma	1633 (99.5)	784 (99.6)	849 (99.3)	0.586
	Follicular carcinoma	9 (0.5)	3 (0.4)	6 (0.7)	
Capsular invasion (%)	No	1145 (69.7)	562 (71.4)	583 (68.2)	0.172
	Yes	497 (30.3)	225 (28.6)	272 (31.8)	
Metastasis number #		3 [2, 7]	3 [1, 5]	4 [2, 8]	< 0.001
Benign lesion (%)	No	696 (42.4)	305 (38.8)	391 (45.7)	0.005
	Yes	946 (57.6)	482 (61.2)	464 (54.3)	
TNM tumor stage (%)	I	1382 (84.2)	670 (85.1)	712 (83.3)	0.336
	II-III	260 (15.8)	117 (14.9)	143 (16.7)	
ATA risk stratification (%)	Low risk	402 (24.5)	303 (38.5)	99 (11.6)	< 0.001
	Intermediate-high risk	1240 (75.5)	484 (61.5)	756 (88.4)	
Tg (ng/mL) #		2.2 [0.6,7.1]	0.9 [0.2, 2.2]	5.7 [2.0, 16.7]	< 0.001
Tg/TSH (ng/mIU) #		24.7 [5.8,83.7]	9.7 [2.3, 25.4]	64.8 [20.3, 192.6]	< 0.001

*TSH* Thyroid stimulating hormone, *Tg* Thyroglobulin, *TNM stage* Tumor-node-metastasis stage of patients, *ER* Excellent response, *NER* Non-excellent response

(%), number (percentage); *, (mean (standard deviation); #, median [first quartile, third quartile]

Statistical tests: (%), the chi-square test; *, the student t-test; #, the Mann–Whitney U test. Statistical significance: *p* < 0.05

#### Analysis of the factors affecting the efficacy of ^131^I radiotherapy

**Fig. 2:**
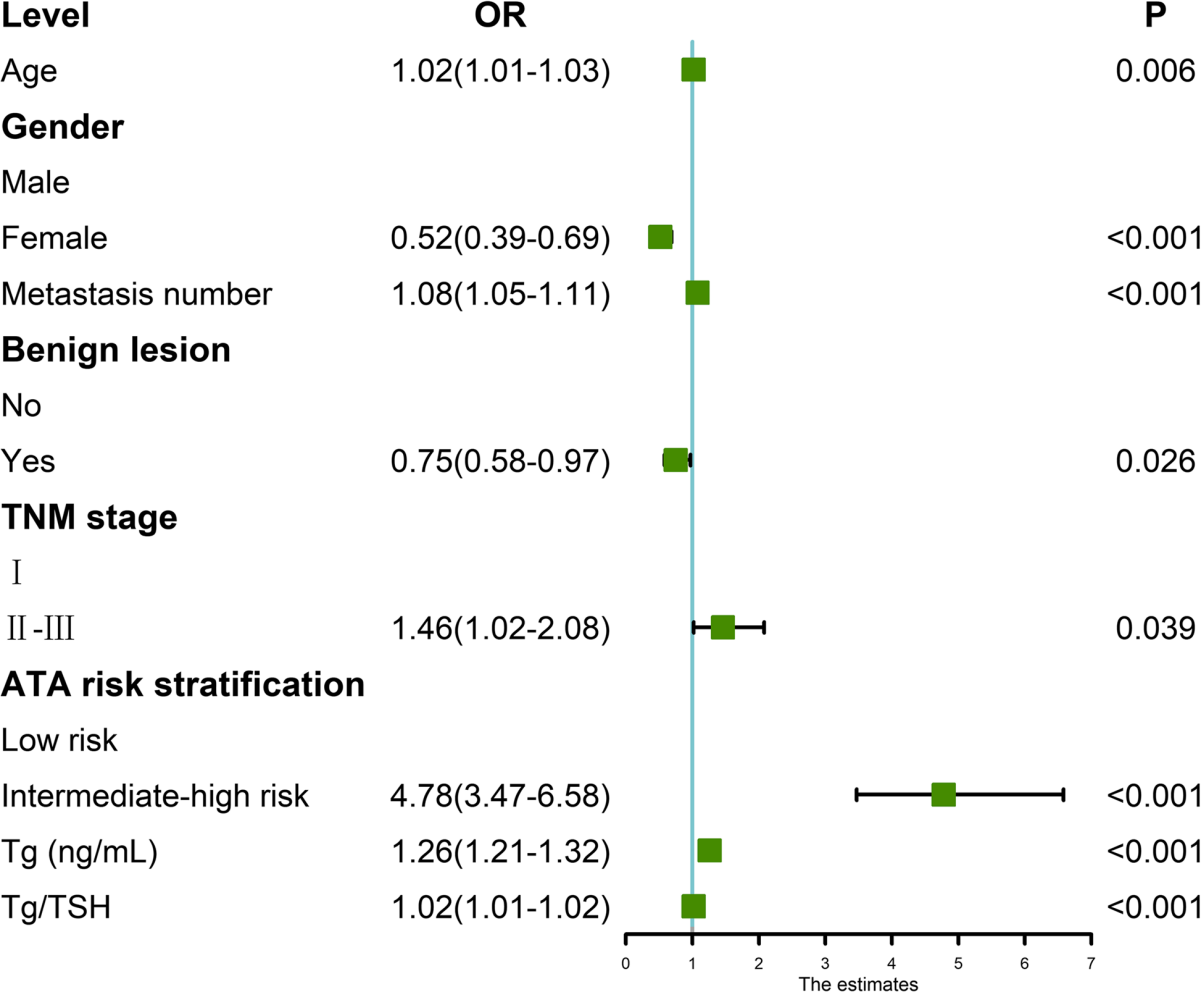
Univariate analysis of the excellent response (ER) of radioactive iodine. *TNM* stage, Tumor-node-metastasis stage of patients; *TSH*, Thyroid stimulating hormone; *Tg*, Thyroglobulin; *OR*, Odds ratio; *CI*, Confidence interval

Univariate analysis showed that the outcome of ^131^I radiotherapy was influenced by age, gender, metastasis number, with or without benign lesion, Tumor-node-metastasis (TNM) stage, ATA risk stratification, pre-treatment Tg level and Tg/TSH level (Fig. 2).

**Fig. 3:**
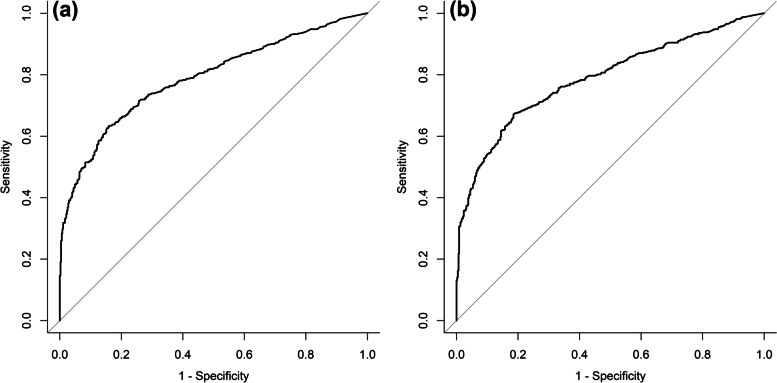
Receiver-operating characteristic (ROC) curves of the thyroglobulin [A: cutoff = 3.40 ng/mL (area under the curve: 0.789) with 63.4% sensitivity and 84.0% specificity] and regarding thyroglobulin/thyroid-stimulating hormone ratio [B: cutoff = 36.03 ng/mIU (area under the curve: 0.788) with 67.2% sensitivity and 81.5% specificity] as predictors of successful radioiodine therapy

According to the ROC curve, the cut-offs for the Tg level was 3.40 ng/ mL [area under the curve (AUC): 0.789] with 63.4% sensitivity and 84.0% specificity, while for Tg/TSH ratio was 36.03 ng/mIU (AUC: 0.788) with 67.2% sensitivity and 81.5% specificity (Fig. 3).

**Fig. 4:**
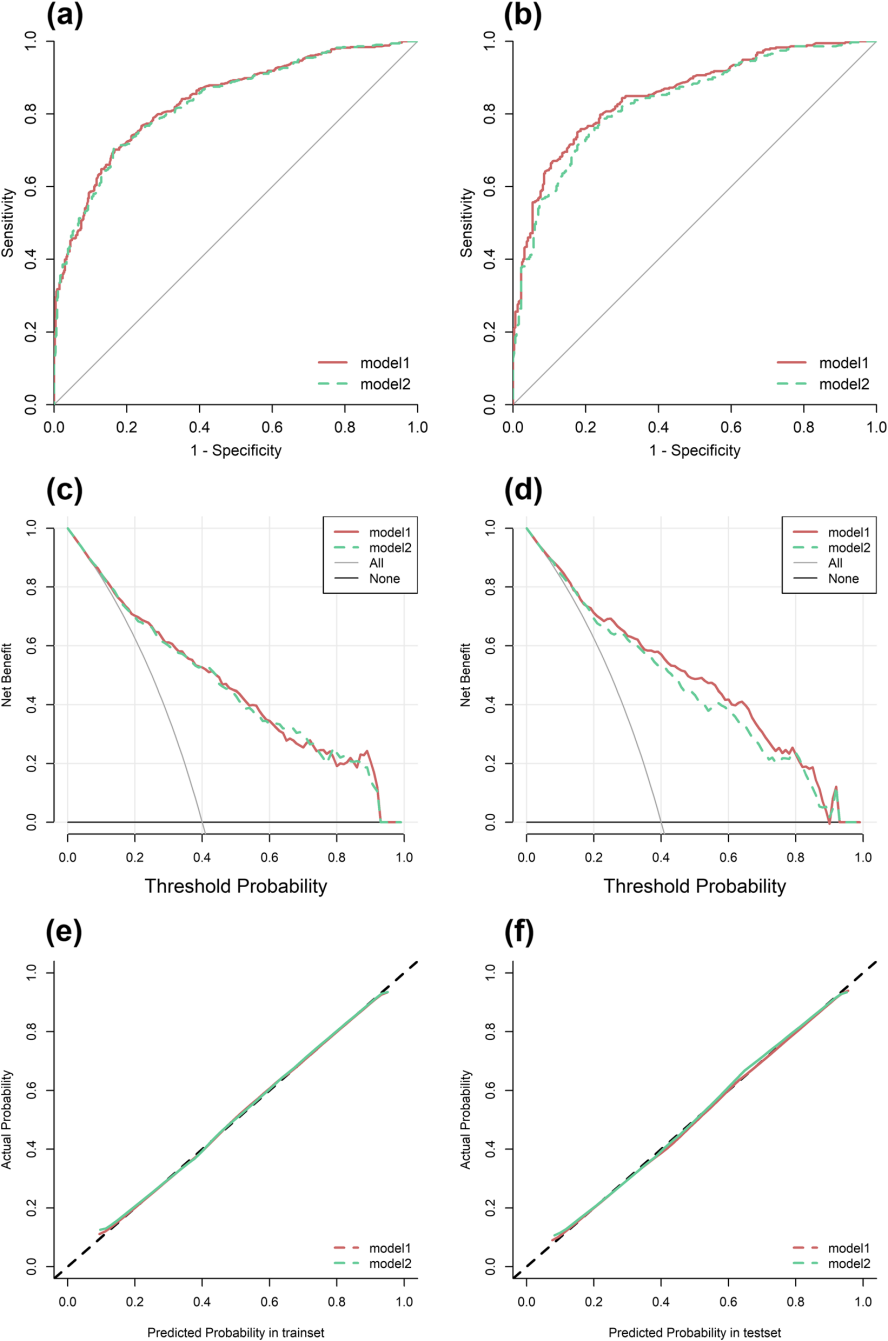
The ROC curves (**A**, **B**), Decision Curves (**C**, **D**), and Calibration Curves (**E**, **F**) of the 2 models. *Model 1*, multiple logistic analysis consisting of age, sex, ATA risk stratification and Tg to the successful radioactive iodine; *Model 2*, multiple logistic analysis consisting of age, sex, ATA risk stratification and Tg/TSH to the successful radioactive iodine; *ROC*, Receiver-operating characteristic; *TSH*, Thyroid stimulating hormone; *Tg*, Thyroglobulin

Age, sex, ATA risk stratification and Tg level were all independent influencers of RAI success in model 1, and age, sex, ATA risk stratification and Tg/TSH level were all independent influencers of RAI success in model 2 (*P* < 0.05) (Table 3). The AUC of model 1 was higher than that of model 2 in both the training set (0.837 vs 0.833) and the testing set (0.854 vs 0.836) (Table 4). Besides, both the ROC curve and decision curve of model 1 in the validation set were significantly higher than that of model 2 (Fig. 4).

**Table 3:** Multiple logistic regression to the successful radioiodine therapy

Indicators	model 1			model 2		
	Estimate	*P* value	*OR* (95%*CI*)	Estimate	*P* value	*OR* (95%*CI*)
(Intercept)	-2.4	< 0.001		-2.169	< 0.001	
Age	0.022	0.002	1.02 (1.01 ~ 1.04)	0.017	0.016	1.02 (1.00 ~ 1.03)
Sex						
male						
female	-0.63	< 0.001	0.53 (0.38 ~ 0.74)	-0.59	0.001	0.55 (0.40 ~ 0.77)
ATA risk stratification						
Low risk						
Intermediate-high risk	1.33	< 0.001	3.78 (2.64 ~ 5.49)	1.362	< 0.001	3.90 (2.73 ~ 5.67)
Tg	0.23	< 0.001	1.26 (1.20 ~ 1.32)			
Tg/TSH				0.017	< 0.001	1.02 (1.01 ~ 1.02)

The factors were assessed by multiple logistic regression and selected by the stepwise procedure, where Tg/TSH was excluded from model 1 and Tg was excluded from model 2

*Tg* Thyroglobulin, *TSH* Thyroid stimulating hormone, *OR* Odds ratio, *CI* Confidence interval

**Table 4:** The ability of models to predict successful radioiodine therapy in the training set and testing set

Indicators	model 1		model 2	
	training set	testing set	training set	testing set
AUC	0.837	0.854	0.833	0.836
Sensitivity	0.702	0.753	0.708	0.747
Specificity	0.834	0.823	0.836	0.792
Optimum cut-off value	0.516	0.498	0.508	0.484

*AUC* Area under the curve, *model 1*, the model consisting of age, sex, ATA risk stratification and Tg level; *model 2*, the model consisting of age, sex, ATA risk stratification and Tg/TSH level

*Tg* Thyroglobulin, *TSH* Thyroid stimulating hormone

## Discussion

Unsuccessful ablation may adversely affect the prognosis of patients with DTC; therefore, predicting the outcome of RAI treatment is important to the treatment and follow-up protocols of patients with DTC. Tg is a glycoprotein that is explicitly produced by thyroid cells and has been demonstrated as a prognosis predictor implying ^131^I therapy response before RAI [12–14]. Since TSH is the trigger factor that directly affects the expression of Tg [15], many have studied the Tg/TSH ratio in addition to Tg to investigate the predictive value for RAI therapy in patients with DTC [16, 17]. Some studies have reported that both Tg and Tg/TSH ratios could be considered predictors of the effects of RAI therapy [9, 18].

In this study, the cutoffs were 3.40 ng/ mL for Tg and 36.03 ng/mIU for the Tg/TSH ratio in predicting successful RAI according to the ROC curve. Though the cutoffs differed from the results of previous studies due to various measurement kits with different sensitivities and display units, this study reached similar findings to some studies that the Tg/TSH ratio and Tg were equally reliable in predicting successful RAI in DTC patients [9, 18]. However, Other studies had different views on the comparison of Tg and Tg/TSH. Some researchers have found that Tg/TSH ratio had a stronger association with ablation outcome than Tg [10]; While others considered that the predictive value of the Tg/TSH ratio was inferior to the prognostic value of Tg alone [19]. The comparison of the Tg and Tg/TSH ratio in predicting the outcome of RAI treatment remained controversial.

To further compare the predictive performance of Tg and Tg/TSH to the response of the RAI therapy, we constructed 2 predictive models and compared them in the training and validation sets respectively. The study showed that in the testing set, the AUC of the predictive model including Tg to predict successful ablation was 0.854 with 75.3% sensitivity and 82.3% specificity, while the AUC of the predictive model including Tg/TSH was 0.836 with 74.7% sensitivity and 79.2% specificity. In addition, the ROC curves and decision curves also showed that the predictive model including Tg performed better in predicting the results of RAI therapy in patients with DTC. Thus, in the present study, the model including Tg had greater predictive accuracy and higher clinical benefit than the model including Tg/TSH. This showed that Tg was a better predictor of the DTC effect than Tg/TSH in the clinical setting.

Compared with other studies, our study included a relatively large number of participants, the outcome of each participant was observed by ourselves, and there was almost no recall bias; In addition, we also conducted a sensitivity analysis by dividing the training set and the validation set to verify the performance of the model, and the research results are convincing. There are several limitations to this study. First of all, the data about TSH tests at several time points could not be analyzed in this study, which was not available in routine practice. Besides, this study had a relatively short follow-up time, and the long-term clinical implication of the Tg and Tg/TSH needs to be assessed by additional prospective studies.

## Conclusion

In conclusion, the present study found that both the Tg and the Tg/TSH ratio could affect the effects of RAI therapy, and the prediction model including Tg performed better than the model including Tg/TSH. Consequently, Tg was more recommended as the factor to predict the efficacy of RAI therapy given its better predictive efficacy in this study.

## Data Availability

The datasets used and/or analysed during the current study are available from the corresponding author upon reasonable request.

## Abbreviations

DTC: Differentiated thyroid cancer
RAI: Radioactive iodine
TSH: Thyroid-stimulating hormone
L-T4: Levothyroxine
ATA: American Thyroid Association
Tg: Thyroglobulin
TgAb: Thyroglobulin antibody
ER: Excellent Response
IDR: Indeterminate Response
BIR: Biochemical Incomplete Response
SIR: Structural Incomplete Response
NER: Non-excellent response
TNM: Tumor-node-metastasis
